# Inference-Optimized AI and High Performance Computing for Gravitational Wave Detection at Scale

**DOI:** 10.3389/frai.2022.828672

**Published:** 2022-02-16

**Authors:** Pranshu Chaturvedi, Asad Khan, Minyang Tian, E. A. Huerta, Huihuo Zheng

**Affiliations:** ^1^Data Science and Learning Division, Argonne National Laboratory, Lemont, IL, United States; ^2^Department of Computer Science, University of Illinois at Urbana-Champaign, Urbana, IL, United States; ^3^National Center for Supercomputing Applications, University of Illinois at Urbana-Champaign, Urbana, IL, United States; ^4^Department of Physics, University of Illinois at Urbana-Champaign, Urbana, IL, United States; ^5^Department of Computer Science, University of Chicago, Chicago, IL, United States; ^6^Leadership Computing Facility, Argonne National Laboratory, Lemont, IL, United States

**Keywords:** gravitational waves, black holes, AI, HPC, GPU-accelerated computing

## Abstract

We introduce an ensemble of artificial intelligence models for gravitational wave detection that we trained in the Summit supercomputer using 32 nodes, equivalent to 192 NVIDIA V100 GPUs, within 2 h. Once fully trained, we optimized these models for accelerated inference using NVIDIA TensorRT. We deployed our inference-optimized AI ensemble in the ThetaGPU supercomputer at Argonne Leadership Computer Facility to conduct distributed inference. Using the entire ThetaGPU supercomputer, consisting of 20 nodes each of which has 8 NVIDIA A100 Tensor Core GPUs and 2 AMD Rome CPUs, our NVIDIA TensorRT-optimized AI ensemble processed an entire month of advanced LIGO data (including Hanford and Livingston data streams) within 50 s. Our inference-optimized AI ensemble retains the same sensitivity of traditional AI models, namely, it identifies all known binary black hole mergers previously identified in this advanced LIGO dataset and reports no misclassifications, while also providing a 3*X* inference speedup compared to traditional artificial intelligence models. We used time slides to quantify the performance of our AI ensemble to process up to 5 years worth of advanced LIGO data. In this synthetically enhanced dataset, our AI ensemble reports an average of one misclassification for every month of searched advanced LIGO data. We also present the receiver operating characteristic curve of our AI ensemble using this 5 year long advanced LIGO dataset. This approach provides the required tools to conduct accelerated, AI-driven gravitational wave detection at scale.

## 1. Introduction

The international network of ground-based gravitational wave interferometers—advanced LIGO (Abbott et al., 2016a,b), advanced Virgo (Acernese et al., 2015; Acernese et al., 2020), and Kagra (Akutsu et al., 2020)—have completed three observing runs, reporting the detection of tens of gravitational wave sources (Abbott et al., 2021b). Within the next decade, these scientific facilities will usher in the era of precision gravitational wave astrophysics, shedding new light into the astrophysical properties of gravitational wave sources, likely formation scenarios, and the nature of the environments where they reside (Abbott et al., 2021d). We have already witnessed the transformational power of gravitational wave astrophysics in fundamental physics, cosmology, chemistry and nuclear physics (Yunes et al., 2016; Abbottet al., 2017a,b; Abbott et al., 2017c, 2021a,c; Mooley et al., 2018; Miller and Yunes, 2019; Tan et al., 2020). These are only a few glimpses of the scientific revolution that may take place within the next decade (Couvares et al., 2021; Kalogera et al., 2021; McClelland et al., 2021; Punturo et al., 2021; Reitze et al., 2021) if we translate the data deluge to be delivered by gravitational wave detectors into the required elements to enable scientific discovery at scale.

Realizing the urgent need to develop novel frameworks for scientific discovery that adequately address challenges brought about by the big data revolution, and acknowledging that many disciplines are undergoing similar transformations thereby increasing the demand on already oversubscribed computational resources, scientists across the world are eagerly developing the next generation of computing frameworks and signal processing tools that will enable the realization of this research program (Huerta et al., 2019).

Over the last few years, it has become apparent that the convergence of artificial intelligence (AI) and innovative computing provides the means to tackle computational grand challenges that have been exacerbated with the advent of large scale scientific facilities, and which will not be met by the ongoing deployment of exascale HPC systems alone (Asch et al., 2018; Huerta et al., 2020). As described in recent reviews (Huerta and Zhao, 2020; Cuoco et al., 2021), AI and high performance computing (HPC) as well as edge computing have been showcased to enable gravitational wave detection with the same sensitivity than template-matching algorithms, but orders of magnitude faster and at a fraction of the computational cost. At a glance, recent AI applications for gravitational wave astrophysics includes classification or signal detection (Gabbard et al., 2018; George and Huerta, 2018a,b; Dreissigacker et al., 2019; Fan et al., 2019; Miller et al., 2019; Rebei et al., 2019; Beheshtipour and Papa, 2020; Deighan et al., 2020; Dreissigacker and Prix, 2020; Krastev, 2020; Li et al., 2020a; Schäfer et al., 2020, 2021; Skliris et al., 2020; Wang et al., 2020; Gunny et al., 2021; Lin and Wu, 2021; Schäfer and Nitz, 2021), signal denoising and data cleaning (Shen et al., 2019; Ormiston et al., 2020; Wei and Huerta, 2020; Yu and Adhikari, 2021), regression or parameter estimation (Gabbard et al., 2019; Chua and Vallisneri, 2020; Green and Gair, 2020; Green et al., 2020; Dax et al., 2021a,b; Shen et al., 2022) Khan and Huerta^1^, accelerated waveform production (Chua et al., 2019; Khan and Green, 2021), signal forecasting (Lee et al., 2021; Khan et al., 2022), and early warning systems for gravitational wave sources that include matter, such as binary neutron stars or black hole-neutron star systems (Wei and Huerta, 2021; Wei et al., 2021a; Yu et al., 2021).

In this article, we build upon our recent work developing AI frameworks for production scale gravitational wave detection (Huerta et al., 2021; Wei et al., 2021b), and introduce an approach that consists of optimizing AI models for accelerated inference, levering NVIDIA TensorRT (NVIDIA, 2021). We describe how we deployed our TensorRT AI ensemble in the ThetaGPU supercomputer at Argonne Leadership Computing Facility, and developed the required software to optimally distribute inference using up to 20 nodes, which are equivalent to 160 NVIDIA A100 Tensor Core GPUs. We quantified the sensitivity and computational efficiency of this approach by processing the entire month of August 2017 of advanced LIGO data (using both Hanford and Livingstone datasets). Our analysis indicates that with our proposed approach, we are able to process these datasets within 50 s using 20 nodes in the ThetaGPU supercomputer at Argonne Leadership Computing Facility. Most importantly, we find that these optimized models retain the same sensitivity of traditional AI models, since they are able to identify all binary black hole mergers in this month-long dataset, while also reporting no misclassifications, and reducing time-to-insight by up to 3*X* compared to traditional AI models (Huerta et al., 2021).

This article is organized as follows. Section Materials and Methods describes the approach we followed to train our AI models, optimize them for accelerated inference, and then combined them to search for gravitational waves as an ensemble. We also describe the advanced LIGO datasets used for training, validation and testing. We summarize our findings in section Results. We outline future directions of work in section Conclusion.

## 2. Materials and Methods

Here, we describe the AI architecture used for these studies, the modeled waveforms and advanced LIGO data used to train and test a suite of AI models. We then describe the procedure to optimize an ensemble of AI models for accelerated AI inference, and the approach followed to deploy this AI ensemble in the ThetaGPU supercomputer to optimally search for gravitational waves in advanced LIGO data at scale.

### 2.1. Modeled Waveforms

In this study, we consider binary black hole mergers, and produce synthetic signals that describe them with the SEOBNRv3 waveform model (Pan et al., 2014) that is available in the open source PyCBC library (Nitz et al., 2021). We densely sample a parameter space that comprises black hole binaries with mass-ratios 1 ≤ *q* ≤ 5, individual spins $s_{\left\{1,2\right\}}^{z}\in \left[-0.8,0.8\right]$, and total mass *M*∈[5M⊙, 100M⊙]. We used a training dataset of over 1,136,415 waveforms, and a validation and testing datasets of over 230k waveforms, sampled at 4096 Hz, to create a suite of AI models in the Summit supercomputer.

### 2.2. Advanced LIGO Data

We used advanced LIGO data available through the Gravitational Wave Open Science Center (Vallisneri et al., 2015). The three data segments we consider have initial GPS times 1186725888, 1187151872, and 1187569664, and are 4,096 s long. Each of these segments include both Hanford and Livingstone data, and do not include known gravitational wave signals.

### 2.3. Data Preparation

We used advanced LIGO data to compute power spectral density (PSDs) estimates using open source software available at the Gravitational Wave Open Science Center. We used these PSDs to whiten both modeled waveforms and advanced LIGO strain data, which are then linearly combined to simulate a wide range of astrophysical scenarios, covering a broad range of signal-to-noise ratios. Following best practices for the training of AI models, we normalized the standard deviation of training data that contain both signals and noise to one. We combined our set of 1,136,415 modeled waveforms with advanced LIGO noise by randomly sampling 1 s long contiguous data samples. To be precise, since we use advanced LIGO data sampled at 4,096 Hz, this means that a 1 s long segment may be described as a set of continuous samples covering the range [*i*_1_, …, *i*_4096_]. In the same vein, another noise realization may be given by the samples [*i*_520_, …, *i*_4596_], etc. This means that in any of the 4,096 s long advanced LIGO data segment we use for training, we could draw 4096 × 4096−4096+1 contiguous, 1 s long noise segments. Since we consider 3 × 4096*s* long advanced LIGO data segments per detector, then it follows that we have at our disposal about 50M noise realizations per detector. Notice, however, that each input that we feed into the net is distinct to each other. This is because each whitened waveform has unique astrophysical parameters, $\left(M,q,s_{1}^{z},s_{2}^{z}\right)$, and is linearly combined with a whitened noise realization that simulates a variety of signal to noise ratio scenarios. On the other hand, we actually find that the number of noise realizations we use for training per detector is given by (# of training iterations × batch size). In our case (# of training iterations → 2, 556, 933) and (batch size → 16). In other words, we use about 40M noise realizations to produce AI models that exhibit strong convergence and optimal performance for gravitational wave detection.

### 2.4. AI Architecture

**Figure 1:**
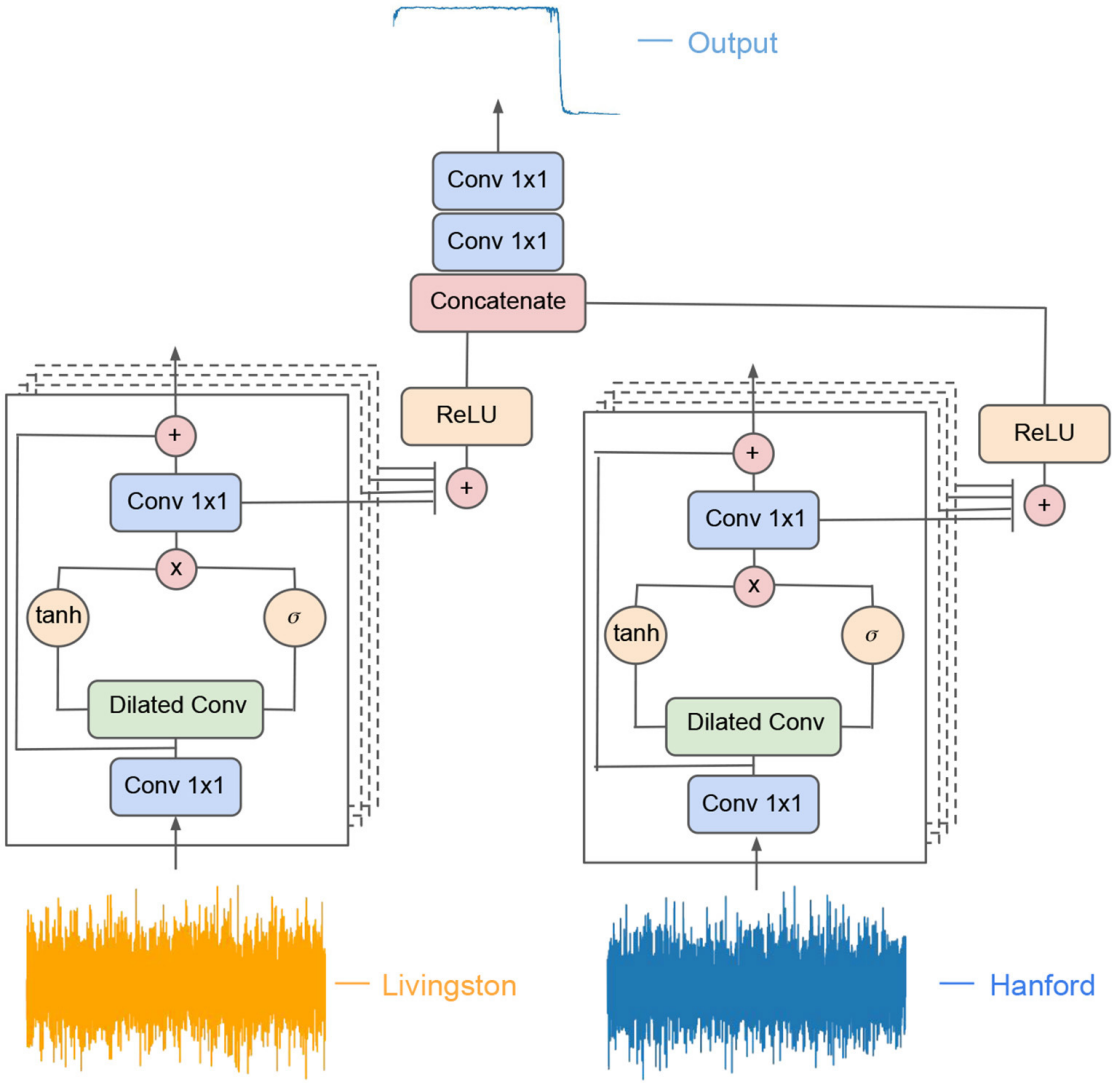
AI architecture. Modified WaveNet model used for gravitational wave detection. Each branch processes concurrently one of the two advanced LIGO data streams—Hanford or Livingston. The output of the two branches is then concatenated and fed into a pair of convolutional layers whose output indicates at each time step whether the input advanced LIGO data contains “noise” or a “waveform”.

We designed a modified WaveNet (van den Oord et al., 2016) architecture that takes in advanced LIGO strain, both from Livingston and Hanford, sampled at 4096Hz. The two outputs of these models (one for each advanced LIGO strain data) are combined and then fed into a set two convolutional layers whose output consists of a classification probability for each time step. The AI architecture used in these studies is depicted in Figure 1.

### 2.5. AI Ensemble Construction

**Figure 2:**
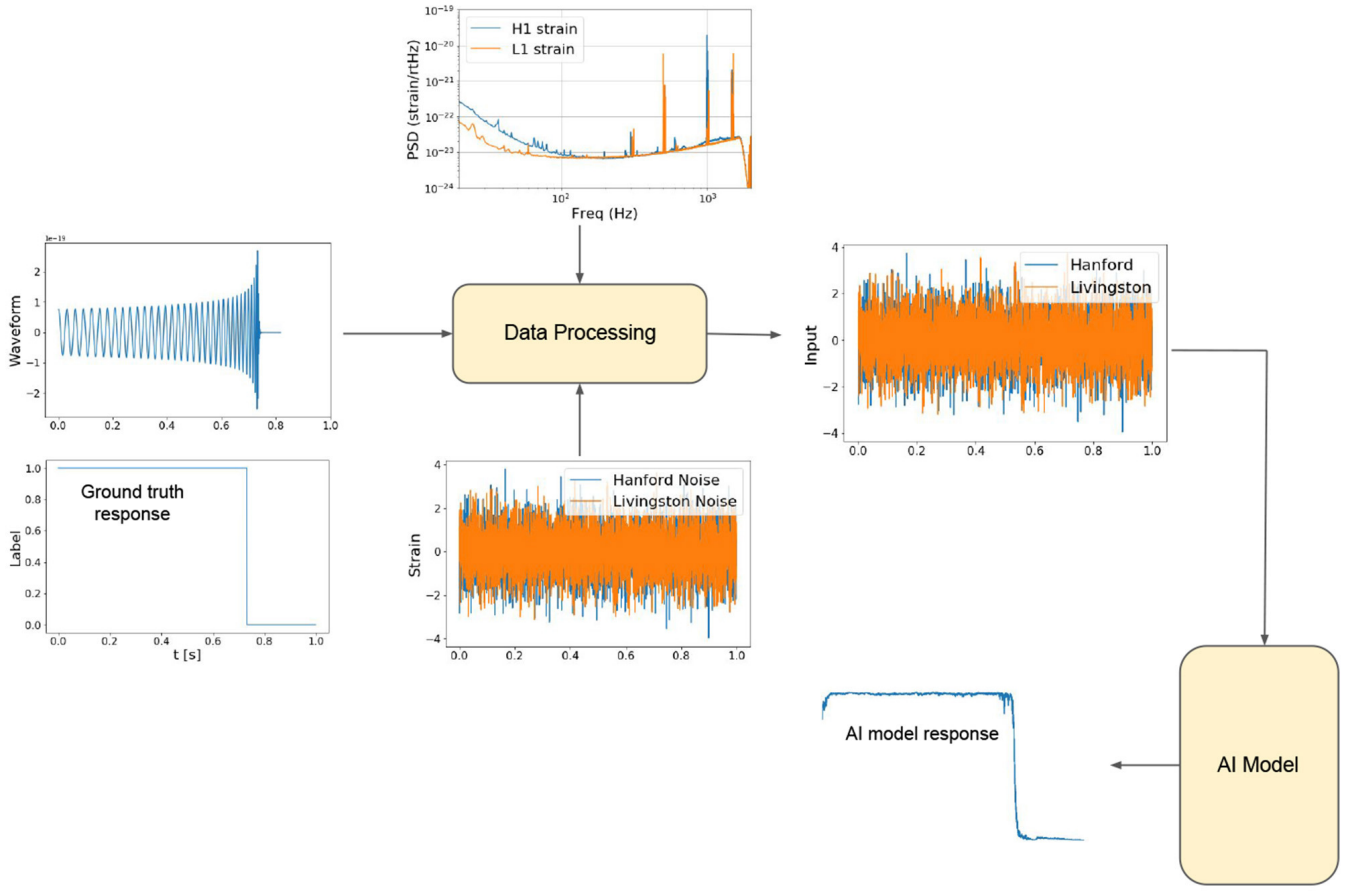
Model creation. Methodology used for data curation, model training, and testing.

During training, the ground-truth labels are curated such that each time step after the merger of a given modeled waveform is classified as “noise”, whereas all the preceding time steps are classified as “waveform”. We used the AI architecture described above and trained a suite of tens of AI models with the Summit supercomputer. We used the same architecture but allowed for random initialization of weights. Each model was trained using 32 Summit nodes, equivalent to 192 NVIDIA V100 GPUs. We then picked a sample of the best ten models and quantified their classification accuracy. We did so by leveraging the feature we encoded in the models to flag the transition between “noise” and “waveform”, which corresponds to the location of the merger of a binary black hole merger. Thereafter, we took the output of these models and post-processed it with the find_peaks algorithm, a SciPy's off-the-shelve tool, to accurately identify the location of these mergers. Finally, we created several combinations of these models and quantified the optimal ensemble that maximized classification accuracy while also reducing the number of false positives in minutes-, hours-, weeks-, and a month-long advanced LIGO strain datasets. This entire methodology, from data curation to model training and testing is schematically presented in Figure 2. Having identified an optimal AI ensemble, we optimized it for accelerated inference using TensorRT.

### 2.6. Optimization With NVIDIA TensorRT

To further reduce time-to-insight with our AI ensemble, we converted our existing AI models, which were originally created in TensorFlow 1 to TensorRT 8 engines. The first step in the conversion process requires us to convert our HDF5 files containing the architecture and weights into the TensorFlow SavedModel format. We then make use of tf2onnx (TensorFlow-ONNX, 2021), an open-source tool for converting SavedModels to the Open Neural Network Exchange (ONNX) format (ONNX Community, 2021). Next, we created a script to describe and build our TensorRT engines and accordingly specified the following parameters: the maximum amount of memory that can be allocated by the engine, which was set to 32 GB (NVIDIA A100 GPUs have 40GB of memory), allowed half-precision (FP16) computation where possible, the input dimensions of the model including the batch size (1024, 4096, 2), the output of the model (1024, 4096, 1), and a flag that allows the built engine to be saved so that the engine will not have to be reinitialized in subsequent runs. TensorRT applies a series of optimizations to the model by running a GPU profiler to find the best GPU kernels to use for various neural network computations, applying graph optimization techniques to reduce the number of nodes and edges in a model such as layer fusion, quantization where appropriate, and more. We found that the TensorRT ensembles allowed us to increase the batch size from 256 to 1024 due to the compressed architecture generated by TensorRT and found an overall average speedup of 3*X* when using the entire ThetaGPU systems for accelerated gravitational wave inference.

### 2.7. Inference-Optimized AI Ensemble Deployment in ThetaGPU

We developed software to optimally process advanced LIGO data using the ThetaGPU supercomputer. We quantified the performance of this approach using 1, 2, 4, 8, 12, 16, and 20 nodes to demonstrate strong scaling. Parallelization was done with mpi4py built on OpenMPI 4. Each GPU, in every ThetaGPU node, acts as one MPI process in our parallel inference script.

## 3. Results

We present three main results: statistical analysis, noise anomaly processing, and computational efficiency of our AI-driven search.

### 3.1. Event Detection

**Figure 3:**
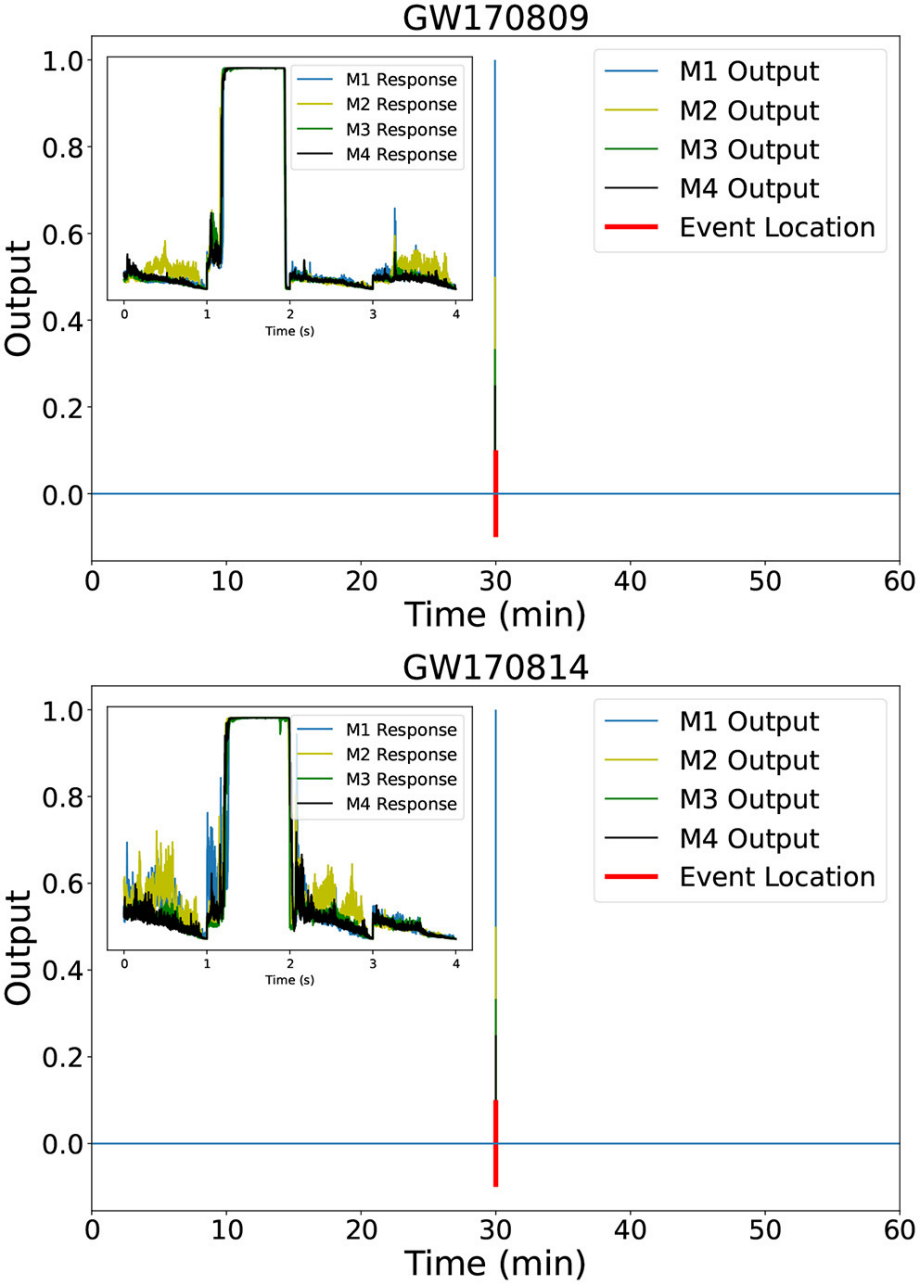
Event detection. Output of the 4 individual AI models in our ensemble upon processing 1 h long advanced LIGO data that contains the events GW170809 (top) and GW170814 (bottom). The insets in both panels show the distinct, sharp response that is common among all AI models when they identify a real signal.

**Figure 4:**
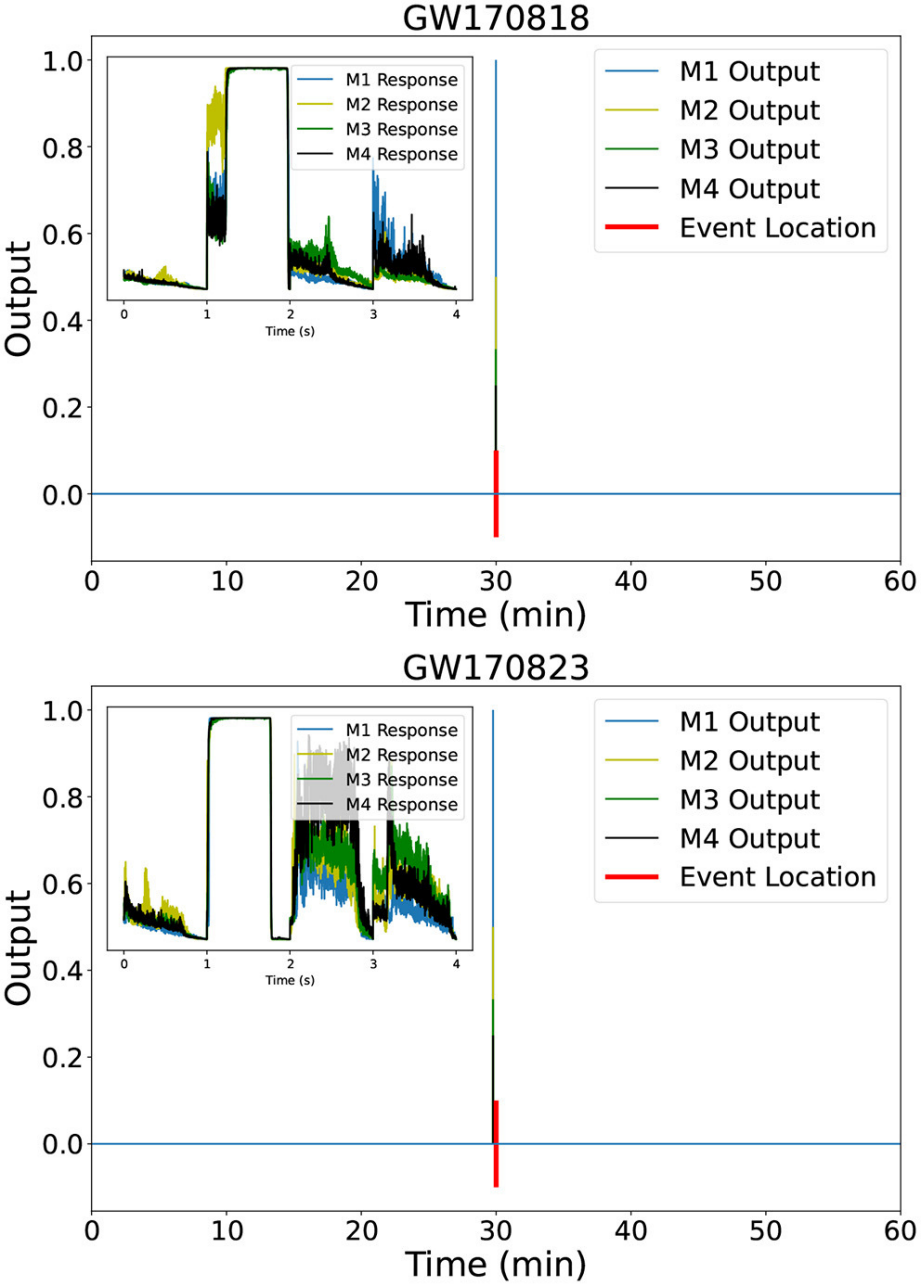
Event detection. As Figure 3, but now for GW170818 (top) and GW170823 (bottom).

We used our inference-optimized AI ensemble to process hours-, days-, weeks-, and a month-long advanced LIGO dataset. We found that this AI ensemble was able to identify all binary black hole mergers reported throughout the second observing run that covered the month of August 2017. Figures 3, 4 show the distinct, sharp response of each of our AI models in the ensemble when they identify real gravitational wave signals. Notice also that the individual models report no other noise trigger of importance within 1 h of data of these four events GW170809, GW170814, GW170818, and GW170823. While Figures 3, 4 show the response of our AI ensemble in the vicinity of these events, we conducted a systematic analysis for all the noise triggers reported by the ensemble upon processing the entire month of August 2017. Triggers that were reported by all AI models in the ensemble, and which were coincident within a time window of 0.5 s were flagged as gravitational wave events. Our analysis only reported four noise triggers of that nature, namely, GW170809, GW170814, GW170818, and GW170823.

### 3.2. Noise Anomaly Processing

**Figure 5:**
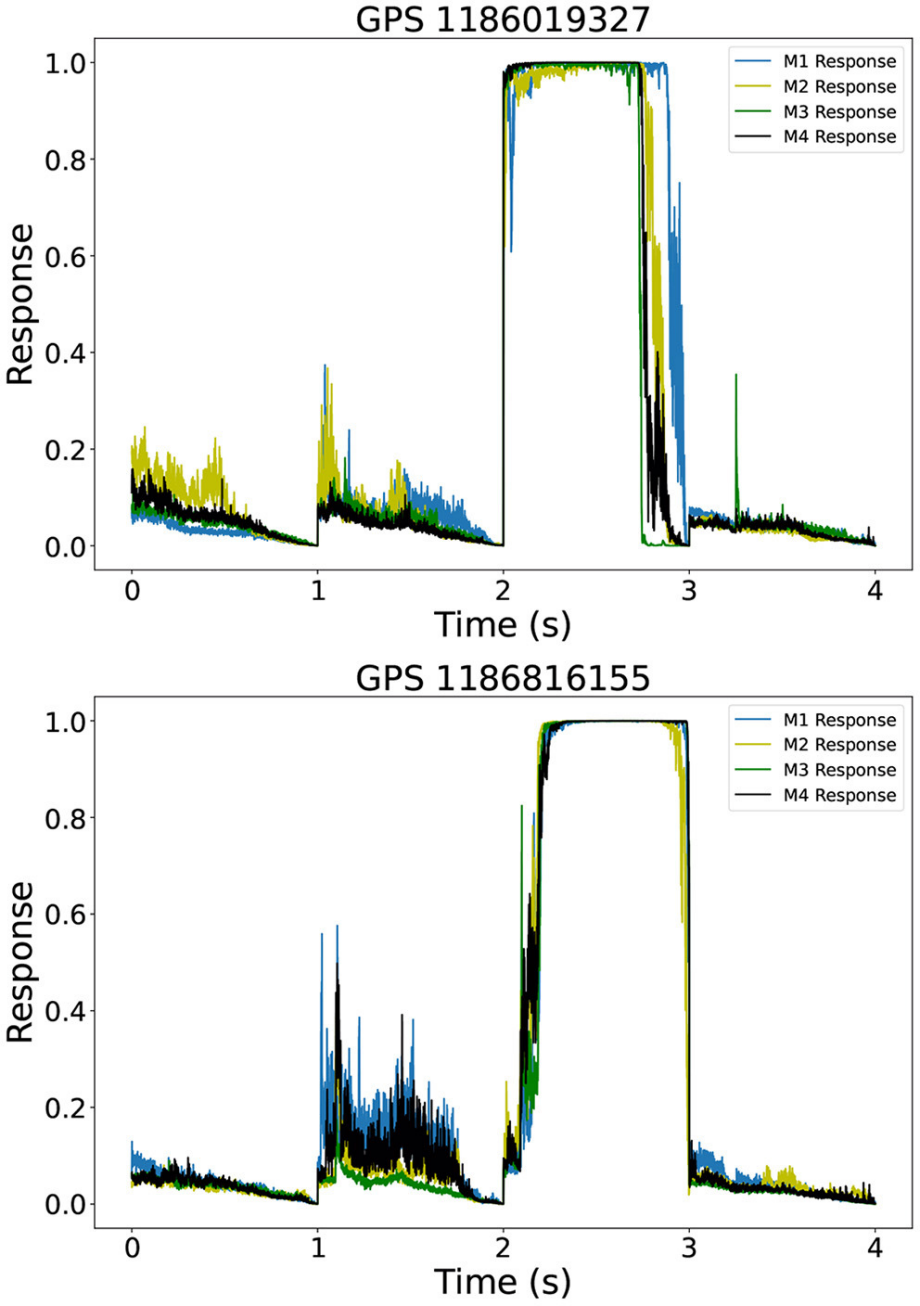
Noise anomalies response of our AI ensemble to real glitches located at GPS times 1186019327 (top) and 1186816155 (bottom).

We quantified the performance of our AI ensemble to discard noise anomalies. To do so, we considered three real glitches in August 2017, namely those with GPS times 1186019327 and 1186816155. In Figure 5, we show the response of our AI ensemble to each of these noise triggers. We notice that the individual AI models in the ensemble do not agree on the nature of these noise triggers, and thus we readily discard them as events of interest. Key features that our find_peaks algorithm utilizes to discard these events encompass the jaggedness and inconsistent widths of these peaks. Since our AI ensemble only identified actual gravitational wave events as relevant noise triggers throughout August 2017, we conclude that our AI ensemble was capable of discarding all other glitches in this 1 month long data batch.

### 3.3. Statistical Analysis

We have quantified the performance of our AI classifiers by going beyond the 1 month worth of data that we used in the previous section for event detection and noise anomaly rejection. To do this, we use time slides to synthetically enhance the month long August 2017 advanced LIGO dataset. Using the approach described in Schäfer and Nitz (2021), we produced datasets that span between 1 and 5 years of advanced LIGO data. Our findings show that our AI ensemble reports, on average, about 1.3 false positives per month. Specifically, we found that the number of false positives for each time-shifted dataset are:

- 1 year worth of data. 22 false positives
- 2 years worth of data. 35 false positives
- 3 years worth of data. 53 false positives
- 4 years worth of data. 68 false positives
- 5 years worth of data. 79 false positives

**Figure 6:**
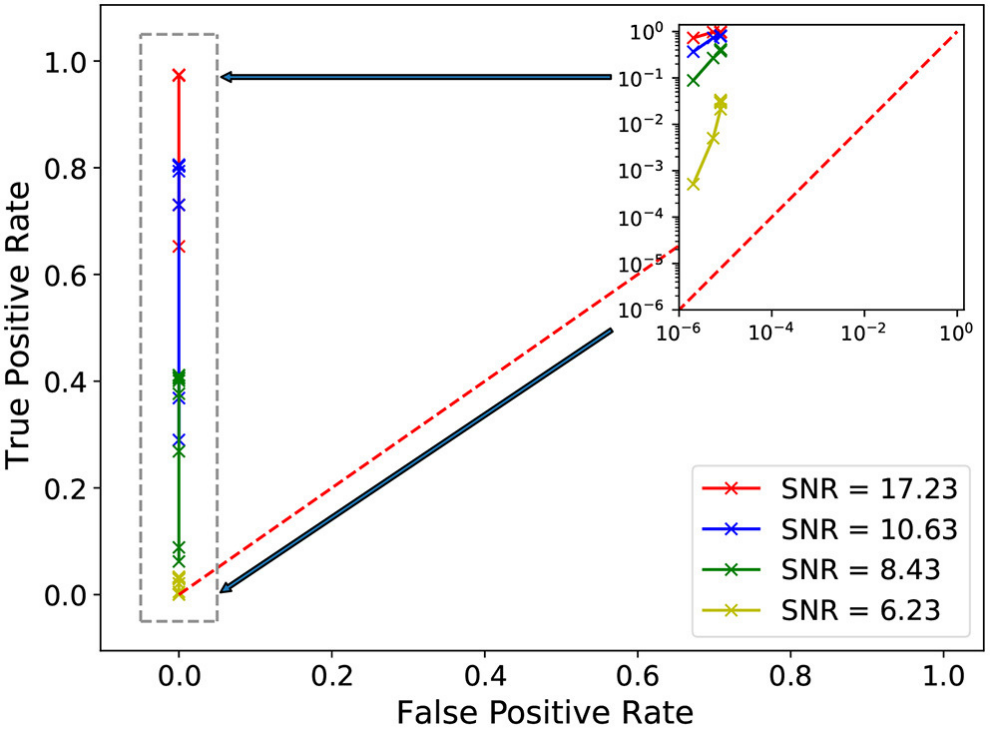
Receiver operating characteristic curve of TensorRT AI ensemble. The output of our inference-optimized AI ensemble is used to estimate the true positive rate with a test set of 237,663 modeled waveforms whitened with advanced LIGO data, and which cover a broad range of signal-to-noise ratios. The false positive rate is computed using a 5 year long time-shifted advanced LIGO dataset. The gray dashed rectangle in the left of this panel is shown in detail in the top right inset.

We have also computed the receiver operating characteristic (ROC) of our AI ensemble, shown in Figure 6. We computed this ROC curve using a test set of 237,663 waveforms that cover a broad range of signal to noise ratios. To compute the ROC curve, we used an automated post-processing script that takes in the output of our AI ensemble, and then uses the find_peaks algorithm to identity peaks whose width is at least 0.5 s long. As shown in Figure 6, our AI ensemble attains optimal true positive rate as we increase the detection threshold, or height in our find_peaks algorithms, between 0 and 0.9998. This plot indicates that our AI ensemble reports, on average, one misclassification per month of searched data. It is worth comparing this figure to other recent studies in the literature. For instance, in Wei et al. (2021b), it was reported that an ensemble of 2 AI models reported 1 misclassification for every 2.7 days of searched data, and more basic AI architectures reported one misclassification for every 200 s of searched advanced LIGO data (George and Huerta, 2018a,b). For completeness, it is worth mentioning that the results we present in Figure 6 differ from those we computed with traditional TensorFlow models in less than 0.01% (Huerta et al., 2021).

It remains to be seen whether adding real glitches to the training stage further improves the detection capabilities of our AI ensemble. We will explore the use of real glitches, e.g., using the catalog curated by the Gravity Spy project (Zevin et al., 2017), to further improve the resilience of our AI models to noise anomalies through adversarial training. Having developed the required framework to time-shift data, in future work we will use a revised version of this AI ensemble to search for gravitational waves over entire observing run datasets. Specific future directions of work involve the production of software and computing methods to post-process the output data of our AI ensemble. At present, our AI ensemble produces about 500GB of output data for every month of searched data. Thus, for the 5 year time-shifted advanced LIGO dataset we considered in this article, we post-processed (5*12*500GB → 30TB) of output data by parallelizing the computing over 1216 AMD EPYC 7742 cores. Thus, while we can now use this method to search for gravitational waves in advanced LIGO data that encompass entire observing run datasets, we will introduce in future work new methods to quantify on the fly the sensitivity of our AI ensemble using hundreds of years worth of time-shifted advanced LIGO data.

### 3.4. Computational Efficiency

**Figure 7:**
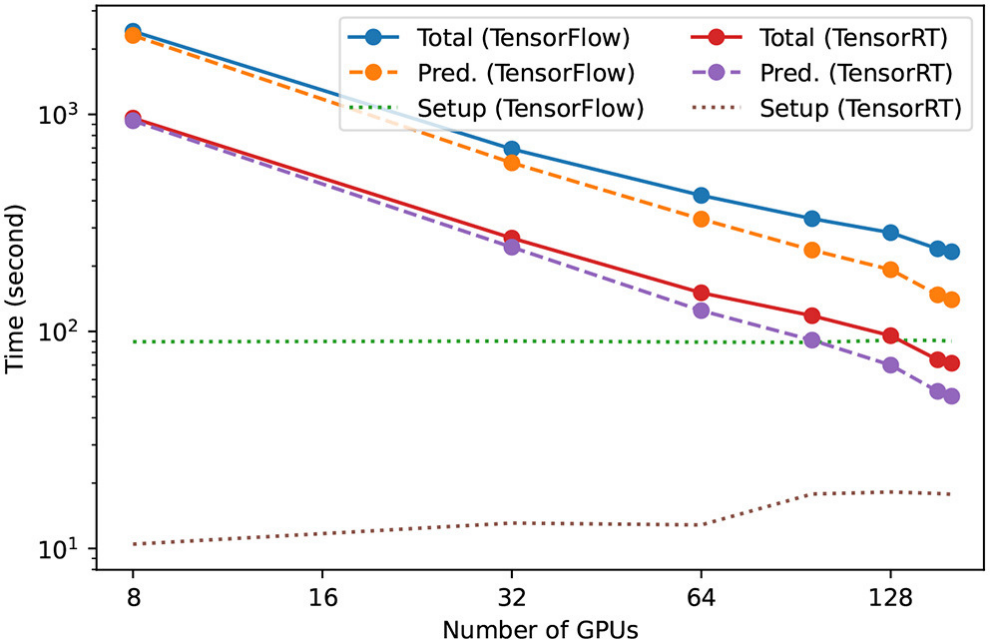
Scaling of accelerated inference in ThetaGPU. TensorRT AI ensembles accelerate gravitational wave detection by 3fold when compared to traditional AI ensembles (labeled as TensorFlow). TensorRT AI ensembles process an entire month of advanced LIGO data, including both Hanford and Livingstone strain data, within 50 s when AI inference is distributed over 160 NVIDIA A100 Tensor Core GPUs in the ThetaGPU supercomputer.

We trained the models in our AI ensemble using distributed training in the Summit supercomputer. Each model was trained using 192 NVIDIA V100 GPUs within 2 h. Thereafter, we distributed the inference using 160 NVIDIA A100 Tensor Core GPUs. Figure 7 presents scaling results as we distributed AI inference in the ThetaGPU supercomputer using both traditional AI models, labeled as TensorFlow, and inference-optimized AI models, labeled as TensorRT. These results show that our TensorRT AI ensemble provides a 3*X* speedup over traditional AI models (Huerta et al., 2021). These results also indicate that the environment setup we used in ThetaGPU optimally handled I/O and data distribution across nodes. It is worth mentioning that these results were reproduced using TensorRT AI ensembles in Singularity containers, and by running our TensorRT AI ensemble natively on ThetaGPU using a suitable Conda environment (Anaconda, 2021). Furthermore, we found that our TensorRT AI ensemble provides additional speedups when we consider larger volume datasets. We will explore the application of this approach for significantly larger datasets in the near future, and will make available these TensorRT AI models through the Data and Learning Hub for Science (Chard et al., 2019; Li et al., 2020b) so that the broader gravitational wave community may harness/extend/improve these AI tools for accelerated gravitational wave data analysis.

**Figure 8:**
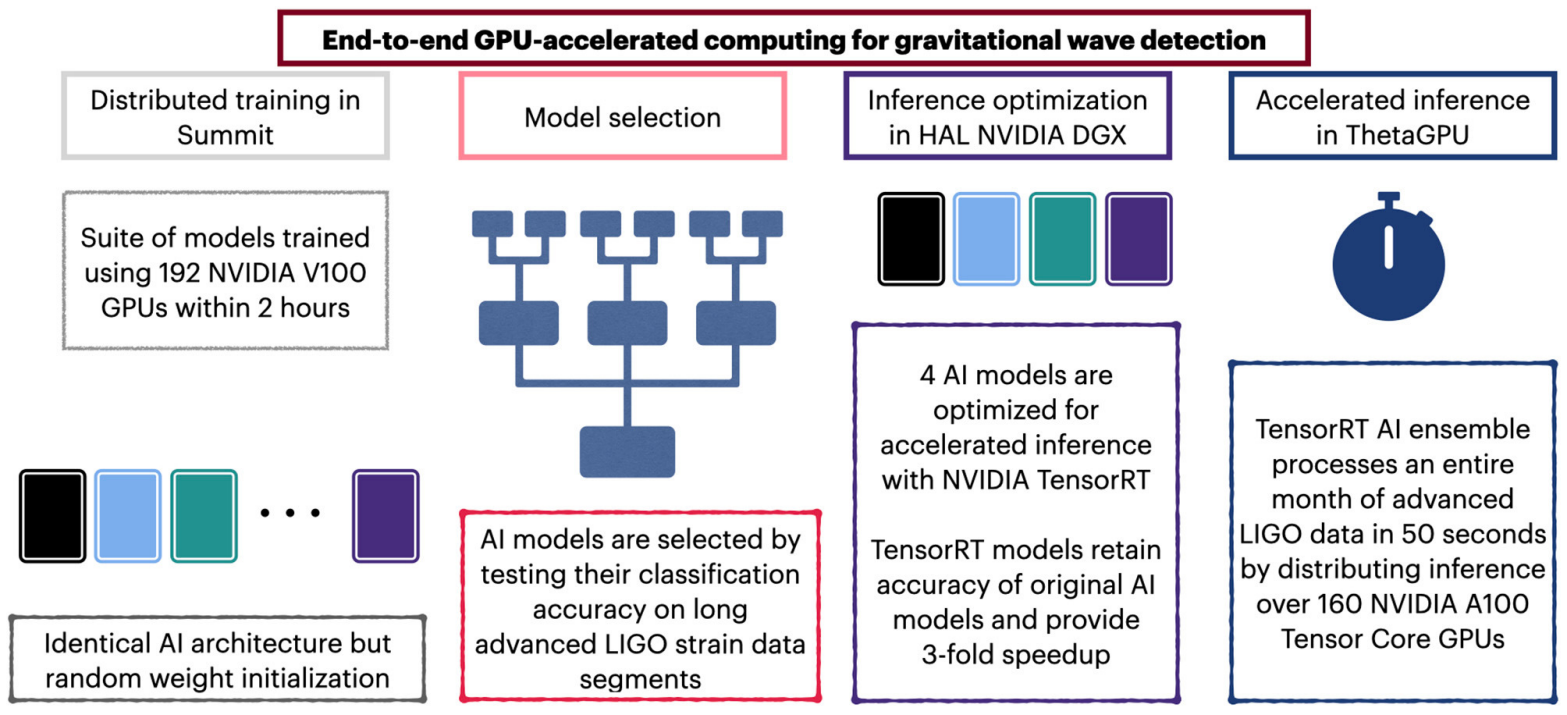
Convergence of AI and HPC. Schematic representation of our methodology to harness disparate HPC platforms and data science tools to create optimal AI ensembles for gravitational wave detection.

This study provides an exemplar that combines HPC systems of different scale to conduct accelerated AI-driven discovery, as shown in Figure 8. We showcase how to optimally use hundreds of GPUs to reduce time-to-insight for training (Summit) and inference (ThetaGPU). It is worth mentioning that we deliberately followed this approach, i.e., using two different machines for training and inference, to quantify the reproducibility and interoperability of our AI ensemble. Another important consideration is that we optimized our AI ensemble with NVIDIA TensorRT using an NVIDIA DGX A100 box at the National Center for Supercomputing Applications. Using this same resource, we containerized our TensorRT AI ensemble using both Docker and Singularity. In brief, our methodology ensures that our AI-driven analysis is reproducible, interoperable and scalable across disparate HPC platforms.

## 4. Conclusion

The first generation of AI models for gravitational wave detection exhibited great promise to accelerate gravitational wave discovery (George and Huerta, 2018a,b), and increase the science reach of gravitational wave astrophysics. Those models provided a glimpse of what may be accomplished if we were able to tap on the computational efficiency and scalability of AI. That vision is gradually coming to fruition by remarkable advances by multiple teams across the world (Huerta et al., 2019; Huerta and Zhao, 2020; Cuoco et al., 2021).

In this article we have described how to combine AI and HPC to accelerate the training of AI models, optimize them for inference, and then maximize their science throughput by distributing inference over tens of GPUs. This line of work has been explored in the context of AI-inference optimized applications for early warning systems. For instance, PyTorch models for AI forecasting of binary neutron star and black hole-neutron star systems were quantized to reduce their size by 4*X* and accelerate their speed 2.5*X* for rapid inference at the edge (Wei et al., 2021a). Furthermore, the combination of TensorRT AI models for data cleaning, and AI models for black hole detection under the umbrella of a generic inference as a service model that leverages HPC, private or dedicating computing was introduced in Gunny et al. (2021). On the other hand, this work is the first in the literature to combine TensorRT AI models for accelerated signal detection with HPC at scale to process 1 month of advanced LIGO strain data from Hanford and Livingston within 50 s using an ensemble of 4 TensorRT AI models. We have not compromised the classification accuracy of our models, and have found that they can identify all four binary black hole mergers previously reported in this data batch, namely, GW170809, GW170814, GW170818, and GW170823, with no misclassifications. When using a time-shifted advanced LIGO dataset that spans 5 years worth of data, we found that our AI ensemble reports 1 misclassification per month of searched data. This should be contrasted with the first generation of AI models that reported 1 misclassification for every 200 s of searched data (George and Huerta, 2018a,b), and the other AI ensembles that reported 1 misclassifications for every 2.7 days of searched data (Wei et al., 2021b).

We are at a tipping point in gravitational wave astrophysics. The number of sources to be detected in the near future will overwhelm available and future computational resources if we continue to use poorly scalable and compute-intensive algorithms. We hope that the AI models we introduce in this paper are harnessed, tested, and further developed by the worldwide community of AI developers in gravitational wave astrophysics. Such an approach will provide the means to transform the upcoming deluge of gravitational wave observations into discovery at scale.

## Data Availability Statement

Publicly available datasets were analyzed in this study. This data can be found here: https://www.gw-openscience.org/eventapi/.

## Author Contributions

EH envisioned this work and led the team to conduct these studies. AK trained the AI models in Summit. MT quantified the performance of AI models and selected those with optimal classification accuracy. PC ported optimal AI ensemble into TensorRT engines. PC and HZ conducted the scaling studies in ThetaGPU. All authors contributed to the article and approved the submitted version.

## Conflict of Interest

The authors declare that the research was conducted in the absence of any commercial or financial relationships that could be construed as a potential conflict of interest.

## Publisher's Note

All claims expressed in this article are solely those of the authors and do not necessarily represent those of their affiliated organizations, or those of the publisher, the editors and the reviewers. Any product that may be evaluated in this article, or claim that may be made by its manufacturer, is not guaranteed or endorsed by the publisher.

## Footnotes

^1^Khan, A., and Huerta, E. A. (under review). AI and extreme scale computing to learn and infer the physics of higher order gravitational wave modes of quasicircular, spinning, non-precessing binary black hole mergers. arXiv preprint arXiv:2112.07669.
